# Quantitative assessment of aeolian desertification dynamics– A case study in north Shanxi of China (1975 to 2015)

**DOI:** 10.1038/s41598-017-11073-8

**Published:** 2017-09-05

**Authors:** Zhanjin Xue, Zuodong Qin, Fangqin Cheng, Guangwei Ding, Hongjian Li

**Affiliations:** 1 Institute of Resources and Environment Engineering, Shanxi University, Taiyuan, Shanxi 030006 China; 2 Institute of Loess Plateau, Shanxi University, Taiyuan, Shanxi 030006 China; 3 Chemistry Department, Northern State University, Aberdeen, SD 57401 USA

## Abstract

Aeolian desertification is one of the serious environmental issues in North Shanxi Province. Accurately assessing aeolian desertification dynamics and its causes is essential to formulate an effective strategy for combating aeolian desertification. Here, we adopted remote sensing (RS) images from four periods (1975, 1990, 2000, and 2015) to classify the intensity of aeolian desertified land (ADL). Four intensity grades (*i.e*., light, moderate, severe, and extremely severe) were categorized based on a series of indices. Then, the RS images were further interpreted coupled with the local climate and socio-economic data to evaluate ADL and its driving force. Results showed that there were 3941.16, 5389.92, 7526.38, and 3752.74 km^2^ of ADL in the above 4 periods, accounting for 28.56%, 39.06%, 54.53%, and 27.19% of the total study area, respectively. ADL experienced three major development stages: slower expansion during 1975–1990 at a rate of 96.58 km^2^/year, rapid expansion during 1990–2000 of 213.65 km^2^/year, and a reversion during 2000–2015 with a net decrease of 251.58 km^2^/year. The ADL development in north Shanxi was a result of mutual interaction between natural factors and human activities. It is also noted that the human activities were identified as the dominant driving force.

## Introduction

Aeolian desertification is one of the most serious eco-environmental and socio-economic problems, especially in the arid, semi-arid, and dry sub-humid areas^1, 2^. It increases land degradation, disrupts the surface water balance, reduces food security, and affects the regional climate^2, 3^. In the past three decades, there has been an increasing appreciation for biological feedbacks^4^, relationships between geomorphic processes and vegetation communities^5^, biogeomorphology^6^, aeolian erosion hazard^7^, and biogeographical patterns^8^ of desertification in the global, especially in the southwestern U.S. (Chihuahuan and Mojave deserts). In China, the area of aeolian desertification amounts to 182.63 × 10^4^ km^2^ by the end of 2014^9^, which is mostly distributed in the northern and northwestern China. Recently, aeolian desertification has attracted the concern of many researchers. The major common outcomes of aeolian research have related desertification not only to its spatiotemporal changes or driving forces^10–15^, but also to its impacts on the environment^16, 17^.

North Shanxi lies on the east of Loess Plateau, and is an important part of farming-pastoral ecotone of northern China. Due to harsh natural conditions, north Shanxi has been suffering from severe aeolian desertification for a long time. North Shanxi is rich in coal and iron resources, which is also included in the Important Base of Energy and Chemical Industry in China. The central government listed Shanxi in the Three-North Shelterbelt Forest Project and the Beijing-Tianjin Sand Source Control Program. At the beginning of the 21st century, the study of aeolian desertification is a sensitive topic due to the conflict between combating aeolian desertification and resource exploration. Over recent decades, researchers have conducted investigations on aeolian desertification in north Shanxi with based on status^18^, distribution^12^, and harmfulness^17, 19, 20^, but such studies did not show the temporal changes of aeolian desertification. Moreover, the relative importance of each specific driving force had not been quantitatively determined in previous investigations.

In the present study, we selected nine counties located in the northern Shanxi as the study area and tried to explore the long-term temporal dynamics of aeolian desertification using multi-spectral satellite images from 1975 to 2015. We then quantitatively identified the causes of aeolian desertification from natural factors (*e.g*., air temperature, precipitation, and gale days) and human factors (*e.g*., population, livestock, and human activities). The results intend to initiate a better-informed decision and develop a more effective strategy in controlling and managing aeolian desertification in north Shanxi^12^. It is expected that results of this study will be beneficial not only to the local level especially in Shanxi Province, but also to the global level.

## Results

### Status of ADL in 2015

**Figure 1:**
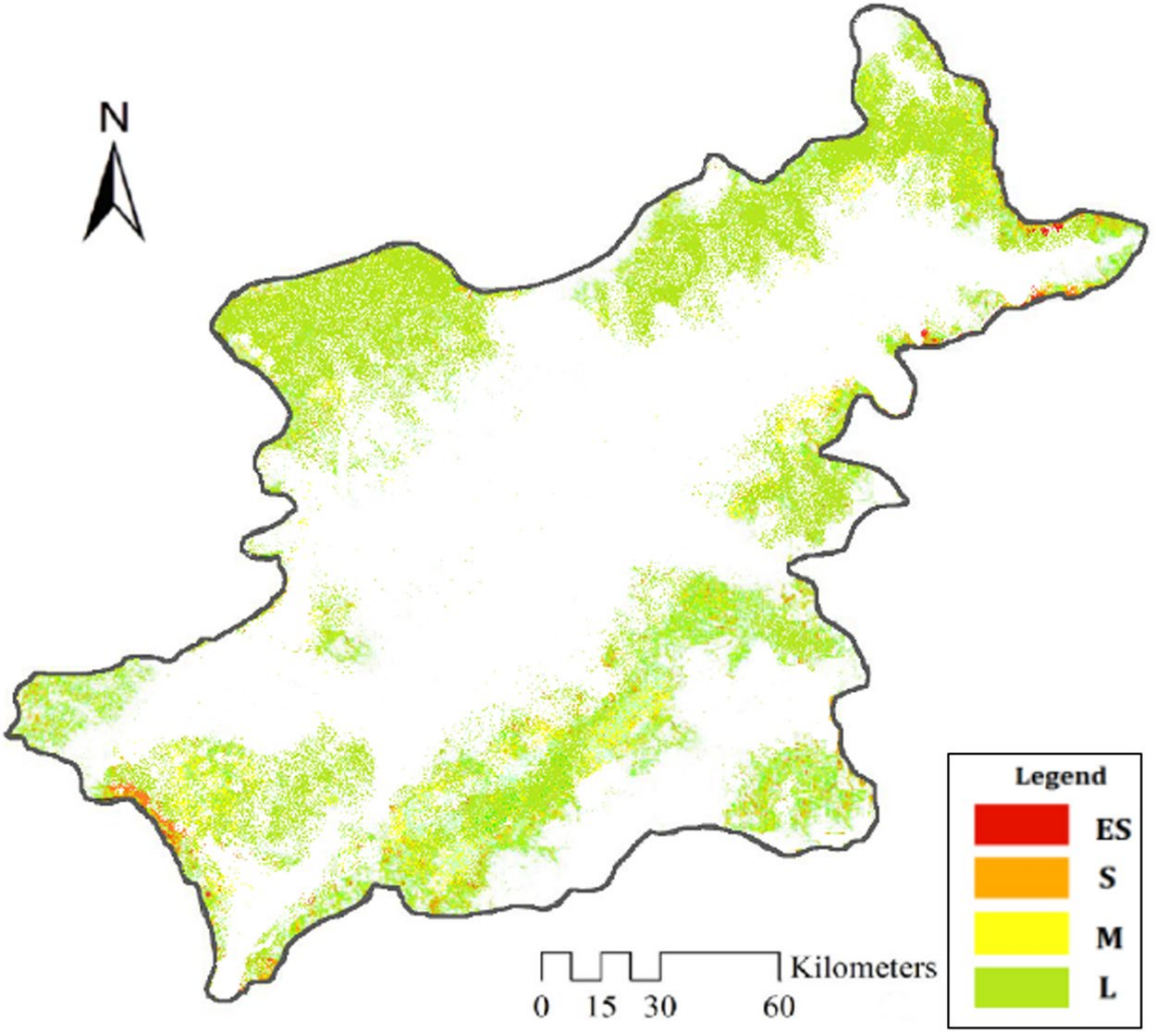
Map of the distribution of ADL in north Shanxi in 2015. The map was plotted using ArcGIS 10.2 (http://www.esri.com/). **L**-Light ADL, M-Moderate ADL, S-Severe ADL, **ES**-Extremely severe ADL. The white areas are the land of no desertification.

Figure 1 shows the aeolian desertified land (ADL) distribution pattern of north Shanxi in 2015. ADL was mainly located over the edge of the study area, where was the higher-elevation terrain, such as piedmonts, alluvial fans, and terraces. The middle of north Shanxi did not distribute the desertified land, its geomorphological domains was basin and river valley. This reveals a weak risk of hazard of wind erosion in low-lying areas or valley fioors^7^. Light ADL was mainly located over the northern regions, such as Tianzhen, Yanggao, and Xinrong, where the ADL area accounted for 68.50% of the total ADL. Moderate and severe ADL exhibited in the southern regions. However, moderate ADL demonstrated a very wide distribution in Shanyin, Yingxian, and Hunyuan. The severe ADL continuously occurred over the southwestern region (Shanyin). Extremely severe ADL was fragmented and was mainly scattered over the northeastern region (Tianzhen). The extent and severity of ADL in the northern region were greater than in the southern region. In total, there were 3752.74 km^2^ of ADL (27.19% of total area) in 2015, of which light, moderate, severe, and extremely severe ADL were 2917.70, 582.16, 147.51, and 105.37 km^2^, accounting for 77.75%, 15.51%, 3.93%, and 2.81%, respectively, of the ADL in the study area (Table 1). The light ADL was the most widely exhibited type.

**Table 1:** Changes in the area of ADL in north Shanxi from 1975 to 2015.

Years	Light		Moderate		Severe		Extremely severe		Total	
	Area(km^2^)	% of ADL	Area(km^2^)	% of ADL	Area(km^2^)	% of ADL	Area(km^2^)	% of ADL	Area(km^2^)	% of total area
1975	3294.04	83.58	457.24	11.60	114.26	2.89	75.62	1.93	3941.16	28.56
1990	4010.66	74.41	947.27	17.57	277.25	5.14	154.74	2.88	5389.92	39.06
2000	5131.78	68.18	1566.92	20.82	481.37	6.39	346.31	4.61	7526.38	54.53
2015	2917.70	77.75	582.16	15.51	147.51	3.93	105.37	2.81	3752.74	27.19

### Temporal Change of ADL

The characteristics of ADL temporal change can be used to explore the relationship between aeolian desertification development and its driving forces^1^. From 1975 to 1990, the area of ADL had been expanded by 1448.76 km^2^, representing a 96.58 km^2^/year increase in the total of ADL since 1975 (Table 2). Thus, this period can be defined as a stage of slower expansion^15^. Four types of ADL expanded in a varying degree. From 1990 to 2000, the area of ADL expanded continuously at a rate of 213.65 km^2^/year, which was faster compared to that during 1975–1990. Therefore, this period can be classified as a stage of rapid expansion^15^. The area of ADL increased in all severity classes. From 2000 to 2015 the area of ADL decreased by 3773.64 km^2^ with a linear decrease rate of 251.58 km^2^/year. This period can be summarized as a stage of reversion. The area in all grades of ADL decreased to a different degree.

**Table 2:** Changes in the area of ADL in various intensity grades.

Periods	Light		Moderate		Severe		Extremely severe		Total	
	Total Change in Area (km^2^)	Change (km^2^/year)	Total Change in Area (km^2^)	Change (km^2^/year)	Total Change in Area (km^2^)	Change (km^2^/year)	Total Change in Area (km^2^)	Change (km^2^/year)	Total Change in Area (km^2^)	Change (km^2^/year)
1975–1990	716.62	47.77	490.03	32.67	162.99	10.87	79.12	5.27	1448.76	96.58
1990–2000	1121.12	112.11	619.65	61.97	204.12	20.41	151.57	15.16	2136.46	213.65
2000–2015	−2214.08	−147.61	−984.76	−65.65	−333.86	−22.26	−240.94	−16.06	−3773.64	−251.58
1975–2015	−376.34	−9.41	124.92	3.12	33.25	0.83	29.75	0.74	−188.42	−4.71

Note: Positive changes represent an increase; negative changes represent a decrease/deposition.

During the 40 years between 1975 and 2015, the total area of ADL dwindled by 188.42 km^2^ and light ADL also shrank by 376.34 km^2^. The other three types of ADL had enlarged by 124.92 km^2^ for moderate, 33.25 km^2^ for severe, and 29.75 km^2^ for extremely severe (Table 2). Therefore, the trend of aeolian desertification in north Shanxi was identified as “an overall reversal but partial deterioration” within the past 40 years^2^.

## Discussion

Aeolian desertification is a complicated process at the level of regional landscape due to the variety of natural factors and anthropogenic factors^12, 16, 21, 22^. In the current project, climate change and human activities were analyzed and quantified with their relative contributions to the dynamics of aeolian desertification^23^.

### Climate factor

**Figure 2:**
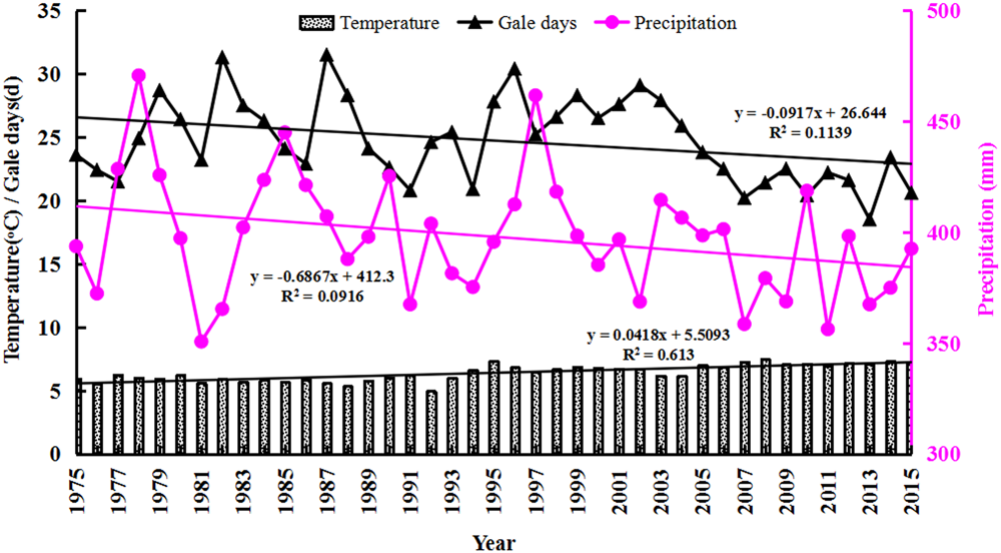
Variations in climate factors in north Shanxi Province from 1975 to 2015.

Temperature, precipitation, and gale days (wind velocity ≥17 m/sec) are three important climate factors affecting aeolian desertification process in north Shanxi. By collecting and analyzing changes of these three variables in this research during the study period (Fig. 2), we found that the average temperature increased markedly with a mean of 0.042 °C/year in our study area, which was greater than the overall rate for China since 1965 (0.022 °C/year) and the global mean rate of increase (0.003–0.006 °C/year) during 1975–2015^15, 24, 25^. The average precipitation showed a larger inter-annual fluctuation with a maximum value of 470.6 mm in 1978 and a minimum value of 350.6 mm in 2011. A significant decreasing rate of 0.716 mm/year was documented from 1975 to 2015. Eventually, the combination of rising temperature and decreasing precipitation resulted in a more arid condition^20^, which intensified vegetation deterioration, wind erosion, and aeolian desertification exacerbation^26^. Meanwhile, the data in Fig. 2 demonstrates that the average gale days fluctuated slightly with a slowly lessening tendency (about 0.014 day/year). However, the gale days were greater from 1975 to 2000 (25.185 day/year) than from 2001 to 2015 (24.680 day/year). There is a cubic relationship between the wind’s erosive power and wind velocity and frequency existed^27^. It is noted that the influence of wind on aeolian desertification was decreasing throughout the study area during 1975–2015 period^23^.

### Human factors

**Figure 3:**
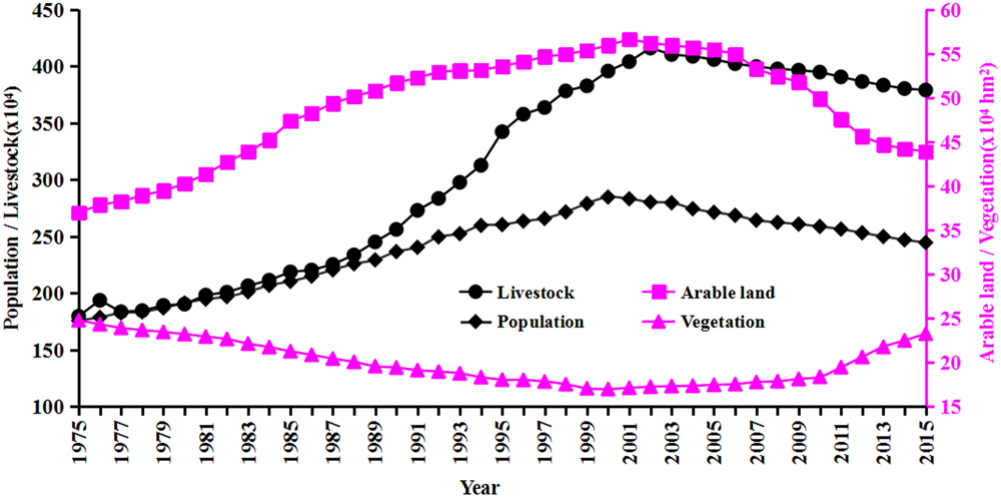
Changes of anthropogenic factors in north Shanxi Province during 1975–2015.

Aside from the above climate driving factors, anthropogenic activities such as population, livestock, vegetation, and arable land were critical important driving factors affecting aeolian desertification dynamics^16^. In north Shanxi Province, the human population increased from 175.6 × 10^4^ in 1975 to 284.6 × 10^4^ in 2000, then decreased steadily to 244.5 × 10^4^ in 2015 (Fig. 3) due to the change of people’s views of family planning. The population density had been 127–206 people/km^2^, which is much higher than the population carrying capacity of 7–22 people/km^2^ in arid and semi-arid regions^17^. The huge population number and excessive population growth rate increased the necessary requirement of land resources and living space, which led to more land for over-cultivation. During the past 40 years, the area of arable land had undergone a tremendous change with an expansion from 36.96 × 10^4^ hm^2^ to 56.61 × 10^4^ hm^2^ during 1975–2001 and reduction from 56.61 × 10^4^ hm^2^ to 43.89 × 10^4^ hm^2^ during 2001–2015, representing a 53.17% increase and 22.47% decrease, respectively. Due to the expansion of arable land and the activation of topsoil, the soil either became eroded or the reclaimed land was subsequently invaded and covered by windblown sand^12^. Although the overall livestock number increased by 2.11 times from 179.6 × 10^4^ to 378.9 × 10^4^ within the past 40 years, it decreased by 8.89% from 415.9 × 10^4^ in 2002 to 378.9 × 10^4^ in 2015. According to our investigation, the actual number of livestock grazing far exceeded the local bearing capacity of grassland (216.9 × 10^4^)^20^, especially in the winter times. Overgrazing and trampling would result in a serious damage to the natural vegetation and the soil structure, thereby accelerating the aeolian desertification processes. Under the influence of the above three proxies of human factors, the vegetation area had an average decrease of 0.31 × 10^4^ hm^2^/year during 1975–2000 and increase of 0.43 × 10^4^ hm^2^/year during 2000–2015. Many people within the study area were living in a poverty-stricken area where unreasonable human activities such as over-cultivation, over-grazing, and over-cutting were very common that led to the vegetation cover decrease. Without the adequate surface protection, the soil was exposed to wind erosion and the fixed sandy land was re-activated into mobile sandy land.

### Identification of driving force

**Figure 4:**
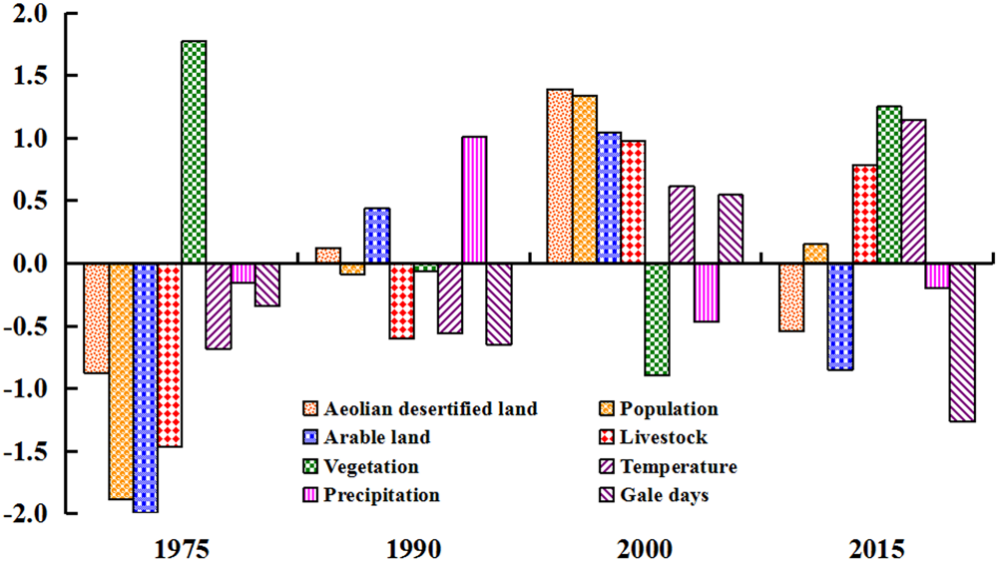
Comparison of ADL in 1975–2015 relative to the proxies of climate and human factors in north Shanxi. All data are normalized.

In this study, the method of standardization and principal component analysis (PCA) were used to analyze the correlation between ADL and both natural and anthropogenic factors. The dynamics of ADL were more consistent with arable land (*x*_2_) and population (*x*_1_), and less consistent with precipitation (*x*_6_) and gale days (*x*_7_) (Fig. 4). The correlation coefficients (*R*) were 0.964, 0.786, 0.352, and 0.245, respectively (Table 3). The trend of ADL change was inconsistent with proxies of vegetation (*x*_4_), livestock (*x*_3_), and temperature (*x*_5_), as correlation coefficients (*R*) were of −0.926, −0.871, and −0.489, respectively.

**Table 3:** Correlation matrix of variable driving changes in ADL.

Variables	Population (*x*_1_)	Arable land (*x*_2_)	Livestock (*x*_3_)	Vegetation (*x*_4_)	Temperature (*x*_5_)	Precipitation (*x*_6_)	Gale days (*x*_7_)	ADL (*Y*)
Population(*x*_1_)	1.000							
Arable land(*x*_2_)	0.789	1.000						
Livestock(*x*_3_)	0.948	0.766	1.000					
Vegetation(*x*_4_)	−0.816	−0.975	−0.827	1.000				
Temperature(*x*_5_)	0.711	0.335	0.703	−0.399	1.000			
Precipitation(*x*_6_)	−0.531	−0.465	−0.442	0.444	−0.338	1.000		
Gale days(*x*_7_)	0.228^*^	0.472	0.233^*^	−0.480	−0.051^*^	−0.062^*^	1.000	
ADL (*Y*)	0.786	0.964	−0.871	−0.926	−0.489	0.352	0.245^*^	1.000

*Signifies statistical difference at *p* < 0.05.

The PCA had reduced the total variance of 7 variables to 2 uncorrelated principal components^28^ such as first principal component and second principal component, the former has the largest possible variance (that is, accounts for as much of the variability in the data as possible). The eigenvalue of the first principal component (4.380) and second principal component (1.257) were both above 1, with co-cumulative reaching 88.528% (å 85%) in north Shanxi (Table 4). This could explain the mechanisms responsible for the ADL dynamics during 1975–2015^29, 30^. Although the variables on the first component showed either as positively or negatively correlated effects, most of them loaded a high absolute value without the large difference. This reflects the versatility and comprehensiveness of the component affecting ADL. The absolute value of factor score coefficients of population (*x*_1_), livestock (*x*_3_), vegetation (*x*_4_), and arable land (*x*_2_) (0.953, 0.937, 0.929, and 0.902, respectively) was more than 0.90. Thus, the first principal component had a close relation with the factors reflecting anthropogenic activity, we may refer to it as a ‘human’ factor, and believe the larger influences of population (*x*_1_) and livestock (*x*_3_) on ADL. Continuous increases of population and livestock exerted a tremendous pressure on the land and water resources^16^, over-cultivation, over-grazing, and over-cutting were very common, which would destroy the surface layer of the soil that protect against wind erosion. For the second principal component, gale days (*x*_7_) and temperature (*x*_5_) had a higher component loading, with the absolute value of factor score coefficients reaching 0.806 and 0.584, respectively. Therefore, the second component had a close relation with climate variation, we may refer to it as ‘natural’ factor, where the gale days (*x*_7_) play a leading role. Windy weathers directly results in the process of removal and transport of soil particles^31^, reactivation of part fixed/semi-fixed aeolian land. Factors affecting erosion rates including soil physical and chemical properties, roughness elements^32^, and surfaces crusts^33^, but wind erosion results in loss of soil thickness and fertility and is a precursor to desertification^31^. The first component and the second component explained 66.572% and 21.956% of the variance in the original 7 variables, respectively. However, the first component was more important. The results confirm the notion that human activity impacts on ADL development had been strengthened in recent decades^28^.

**Table 4:** Eigenvalues and cumulative of components matrix of driving factors in ADL^a^.

Variable^b^	*x*_1_	*x*_2_	*x*_3_	*x*_4_	*x*_5_	*x*_6_	*x*_7_	Eigenvalues	Variance/%	Cumulative/%
Component1	0.953	0.902	0.937	−0.929	0.648	−0.590	0.388	4.380	66.572	66.572
Component2	−0.181	0.299	−0.160	−0.267	−0.584	0.220	0.806	1.257	21.956	88.528

^a^Extraction method: principal component analysis. ^b^Rotation converged in 3 iterations. Varimax with Kaiser normalization.

## Methods

### Study Area

**Figure 5:**
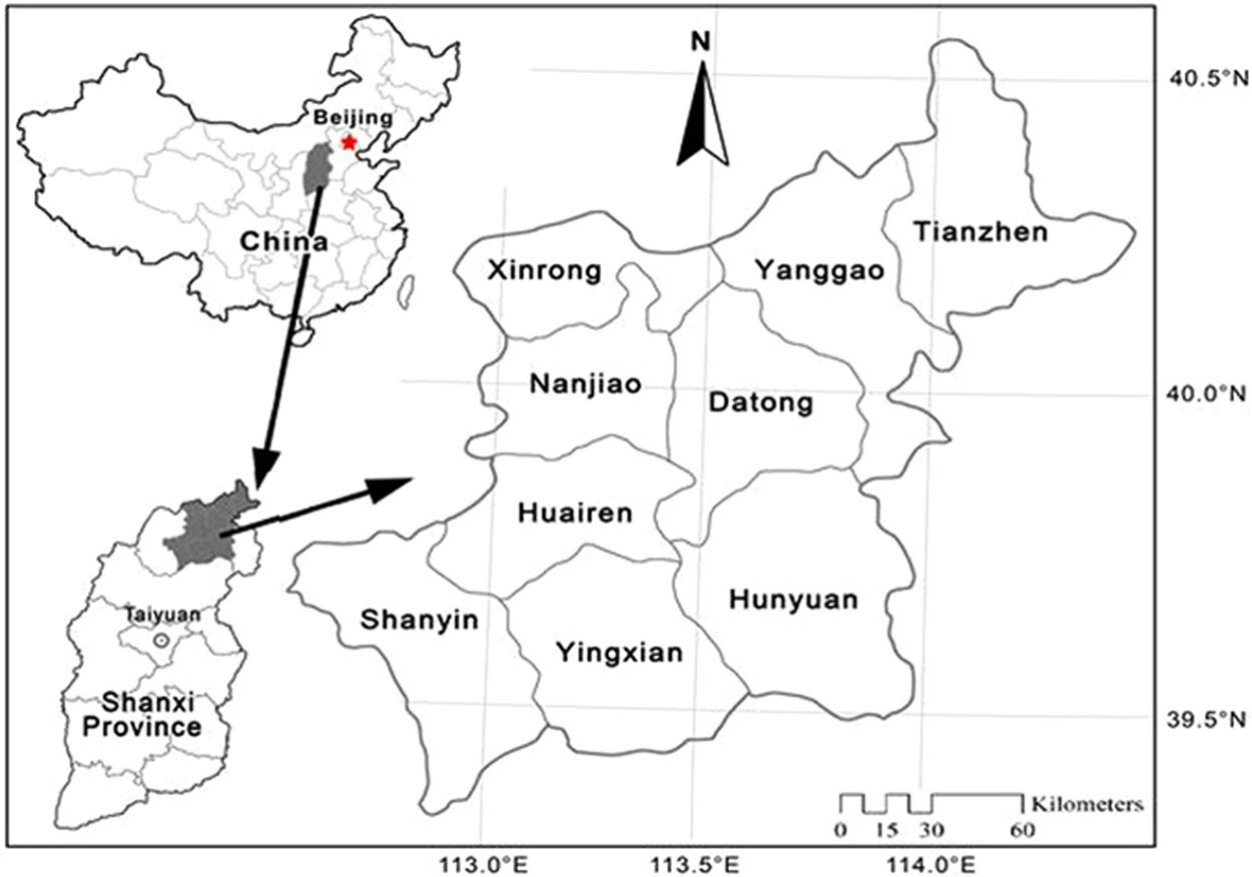
Location of the study area. The map was plotted using ArcGIS 10.2 (http://www.esri.com/).

The study area is located at longitudes 112°25′ E to 114°32′ E, latitudes 39°11′ N to 40°43′ N (Fig. 5). It covers about 1.38 × 10^4^ km^2^ and includes nine counties (Tianzhen, Yanggao, Xinrong, Nanjiao, Datong, Hunyuan, Huairen, Yingxian, and Shanyin). The terrain of the region is mostly within Datong basin. It is surrounded by the Hongtao Mountains to the west, the Taihang Mountains to the east, the Hengshan Mountains to the south, and the Great Wall to the north. Elevations vary between 1200 and 2400 m above sea level. The region is characterized by a temperate continental monsoon climate, *i.e*., with dry and cold winter, arid and windy spring^17^. The mean annual air temperature ranges from 4.5 to 8.0 °C. The average annual precipitation varies from 350 to 460 mm, in which nearly 75% to 80% of the rainfall occurs during June-September. The annual potential evaporation is between 1780 and 1950 mm, which is 4.2 to 5.1 times that of the average annual precipitation. The mean annual wind velocity was more than 4.2 m/sec with prevailing NW/NNW winds in winter and spring, SE winds in summer and autumn. The mean number of gale days is more than 25 days (mainly in the period from March to May). The study area is a transition zone between warm temperate deciduous forest and temperate grassland^17^. The dominant species are *Hippophae rhamnoides* Linn., *Ostryopsis davidiana* Decne., *Caragana korshinskii* Kom., *Bothriochloa ischaemum* (L.) Keng, *Rosa xanthina* LindlI, and *Artemisia argyi* Levl. Across the region, zonal soil mainly includes Calci-Ustic Isohumosols, Hapli-Ustic Argosols, Haply-Udic Cambosols, and Loessi-Orthic Primosols^12, 34^, the mechanical composition of these soil is predominantly loose and sandy material, the clay fraction (<0.002 mm) was under 20%, which to be extremely easy to cause wind erosion.

The major economy for the local residents is dominated by rain-fed agriculture and mining industry. During the past few decades, the growth of rural population and urban expansion have deteriorated the vegetation and exposed the soil^35^. These factors, together with adversely natural conditions (*e.g*. arid climate, windy weather, and sandy soil), made aeolian desertification being a serious environmental problem in the northern Shanxi.

### ADL Classification System

**Figure 6:**
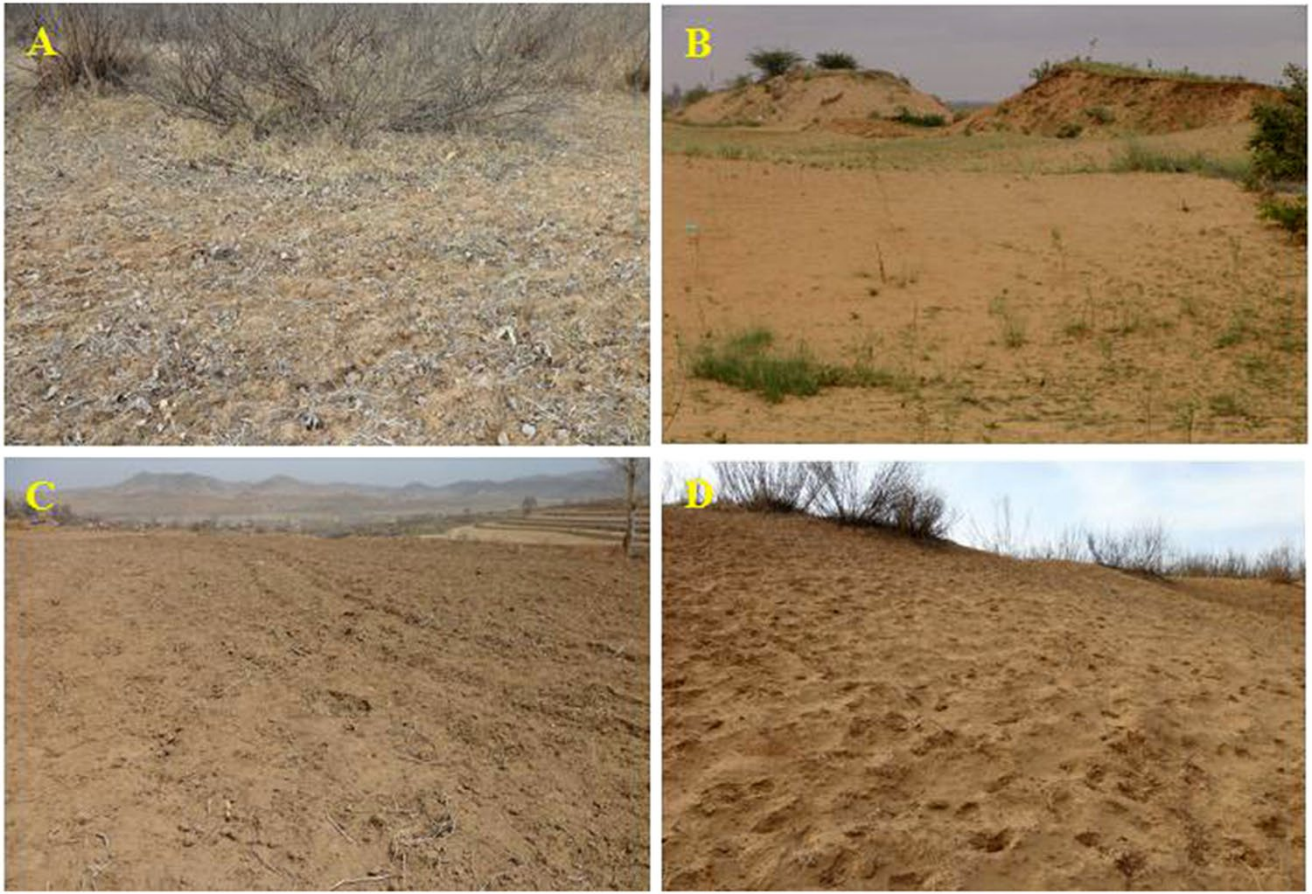
Main types of ADL in the northern Shanxi. (Images were taken by Z.J. Xue). (**A**–**D**) correspond to the types of ADL in Table 5, A-Light ADL, B-Moderate ADL, C-Severe ADL, D-Extremely severe ADL.

Some major factors (*i.e*., dispersed, patchy vegetation cover, and sand sheets) were considered as the visual indicators of environmental changes and the main landscape characteristics of aeolian desertification^12, 36^. Referring to the classification criteria proposed in previous studies^1, 10, 28, 37–40^, field investigations, and analysis of Landsat images, the proportions of the total area covered by mobile sand, wind-eroded areas, vegetation cover, surface temperature retrieving, and texture analysis were selected to describe the severity of aeolian desertification in the current project^16^. The aeolian desertification of the study area was identified in four intensity categories: light, moderate, severe, and extremely severe, according to the criteria shown in Table 5 and Fig. 6 ^12^.

**Table 5:** Classification of ADL in the northern Shanxi.

Categories	Landscape characteristics	Tone and texture of remote sensing images
Light	Mobile sand is below 5%, blowout appears on windward slope of sand dunes, and mobile sand is speckled. Vegetation cover exceeds 60%, most parts of the area still resemble the original landscape.	Light red, dotted by darker red, and coarse and partially bare surface were spotted in the images.
Moderate	Mobile sand is 5–25%, and appears on windward slopes of shrub sand dunes and flats between sand dunes. Vegetation cover is 30–50%, but vegetation is interspersed with sand sheets or wind-eroded areas.	Irregular blocks and the shapes of sand dunes are clearly identified.
Severe	Mobile sand is 25–50%, and sand dunes are in a half-shifting state. A large number of sandy pioneers’ plants appear, and vegetation cover is 10–30%.	Irregular brownish yellow or yellow white surface, dotted shrubs can be recognized.
Extremely severe	Mobile sand is higher than 50%, sand dunes in a shifting state. There is little or no vegetation (*i.e*., vegetation cover is below 10%).	Yellow or white surface, or no mobile sand dunes or sand ridges can be documented. A wavy texture feature is recorded.

### Data Acquisition and Processing

Remote sensing (RS) and geographic information system (GIS) combined techniques have played an important role in the exploration of temporal-spatial variability of aeolian desertification^14, 41, 42^. RS data used in this study included 8 scenes (2 scenes for each period) of four sets of Landsat Multispectral Scanner (MSS) and Thematic Mapper (TM)/Enhanced Thematic Mapper (ETM) images in 1975, 1990, 2000, and 2015. The identification of RS images and the data collection were to apply the multi-spectral data combination technology and image mosaic technology in v9.3 of the Erdas Imagine software (http://www.hexagongeospatial.com). We acquired MSS images for the 1975 data, TM images for the 1990 and 2015 data, and ETM images for the 2005 data, with a spatial resolution of 80-m (MSS) and 30-m (TM/ETM). The MSS image was obtained from Shanxi Center of Geographical Information, and the TM/ETM images were downloaded from the site http://glovis.usgs.gov. All cloud-free/little cloud (less than 5%) images were obtained from July to September because vegetation typically reaches its maximum growth. The control points were selected in the topographic map. Landsat images were corrected by the quadratic polynomial resample method^30^ to ensure the correction precision within 0.5 pixels, and rectified by the cubic convolution resample method^30^ to set up the Unified Albers Coordinate Projection System, with a superposition analogy of RS images to analyze and extract ADL information. The GIS techniques allow the use of information available in digital format that is compatible to and implementable in different databases (ArcGIS 10.2 (http://www.esri.com/))^43^. Under the support of Erdas Imagine v9.3 and ArcGIS 10.2, the multi-spectral directed classification and field investigations were used to obtain ADL data by the visual interpretation of RS images according to Table 5. The interpretation accuracy was over 95% according to *in-situ* field survey results^44^.

Local meteorological and socio-economic data were collected and analyzed to explore the mechanism and processes of aeolian desertification. Meteorological data, including mean annual precipitation, mean annual temperature, and mean annual gale days from 1975 to 2015, were obtained from 7 meteorological stations in and near the study area. As the proxies of socio-economic data between 1975 and 2015, we chose population, livestock numbers, arable land area, and forest coverage, which were collected from Datong Bureau of Statistics of Shanxi, Shuozhou Bureau of Statistics of Shanxi, and www.stats-sx.gov.cn. Thematic maps, including DEM maps, soil maps, and land use maps, were also used as the supplementary data sources.

## Analytical Methods

In other regions in the northern China, the temporal changes of ADL are expressed in the following equation^14^.

1 $$V=({S}_{1}-{S}_{2})/T$$

where *V* is the temporal dynamic degree (dimensionless) of ADL, *S*_1_ is the area (km^2^) for the year, *S*_2_ is the ADL area (km^2^) for the previous year, and *T* is the time interval (year) between *S*_1_ and *S*_2_.

The method of standardization and PCA were used to identify the correlation between ADL and the factors affecting desertification. The standardization formula has been described by Shen *et al*.^28^.

2 $$z=(x-\mu )/\sigma $$

where z is the standardized data in dimensionless, *x* is the original data in units of above 7 variables, *μ* is the arithmetic mean of original data in units of above 7 variables, and *σ* is the standard deviation in units of above 7 variables. Then, we conducted PCA to determine the contribution of the natural and human factors to ADL development, using the Social Sciences software (SPSS 19.0).
